# Improved Analysis of COVID-19 Influenced Pneumonia from the Chest X-Rays Using Fine-Tuned Residual Networks

**DOI:** 10.1155/2022/9414567

**Published:** 2022-06-16

**Authors:** Amel Ksibi, Mohammed Zakariah, Manel Ayadi, Hela Elmannai, Prashant Kumar Shukla, Halifa Awal, Monia Hamdi

**Affiliations:** ^1^Department of Information Systems, College of Computer and Information Sciences, Princess Nourah bint Abdulrahman University, P.O.Box 84428, Riyadh 11671, Saudi Arabia; ^2^College of Computer and Information Sciences, King Saud University, P.O.Box 51178, Riyadh 11543, Saudi Arabia; ^3^Department of Information Technology, College of Computer and Information Sciences, Princess Nourah bint Abdulrahman University, P.O.Box 84428, Riyadh 11671, Saudi Arabia; ^4^Department of Computer Science and Engineering, Koneru Lakshmaiah Education Foundation, Vaddeswaram, Guntur, Andhra Pradesh, India; ^5^Kwame Nkrumah University of Science and Technology, Kumasi, Ghana; ^6^Department of Electrical and Electronics Engineering, Tamale Technical University, Tamale, Ghana

## Abstract

COVID-19 has remained a threat to world life despite a recent reduction in cases. There is still a possibility that the virus will evolve and become more contagious. If such a situation occurs, the resulting calamity will be worse than in the past if we act irresponsibly. COVID-19 must be widely screened and recognized early to avert a global epidemic. Positive individuals should be quarantined immediately, as this is the only effective way to prevent a global tragedy that has occurred previously. No positive case should go unrecognized. However, current COVID-19 detection procedures require a significant amount of time during human examination based on genetic and imaging techniques. Apart from RT-PCR and antigen-based tests, CXR and CT imaging techniques aid in the rapid and cost-effective identification of COVID. However, discriminating between diseased and normal X-rays is a time-consuming and challenging task requiring an expert's skill. In such a case, the only solution was an automatic diagnosis strategy for identifying COVID-19 instances from chest X-ray images. This article utilized a deep convolutional neural network, ResNet, which has been demonstrated to be the most effective for image classification. The present model is trained using pretrained ResNet on ImageNet weights. The versions of ResNet34, ResNet50, and ResNet101 were implemented and validated against the dataset. With a more extensive network, the accuracy appeared to improve. Nonetheless, our objective was to balance accuracy and training time on a larger dataset. By comparing the prediction outcomes of the three models, we concluded that ResNet34 is a more likely candidate for COVID-19 detection from chest X-rays. The highest accuracy level reached 98.34%, which was higher than the accuracy achieved by other state-of-the-art approaches examined in earlier studies. Subsequent analysis indicated that the incorrect predictions occurred with approximately 100% certainty. This uncovered a severe weakness in CNN, particularly in the medical area, where critical decisions are made. However, this can be addressed further in a future study by developing a modified model to incorporate uncertainty into the predictions, allowing medical personnel to manually review the incorrect predictions.

## 1. Introduction

COVID-19 belonged to the “coronavirus” (CoV) lineage when it was discovered for the first time in December of this year. The World Health Organization (WHO) reclassified the “severe acute respiratory syndrome coronavirus 2” (SARS-CoV-2) to COVID-19 in February of this year. On March 11, 2020, the World Health Organization (WHO) announced COVID-19 as a pandemic, and it was designated as a Public Health Emergency of International Concern [1]. Fever, coughing, and respiratory sickness (similar to the flu) may occur in some people exposed to the COVID-19. In contrast, no symptoms will be present in many others [2]. Pneumonia, difficulty breathing, organ failure, and even death are potential indications of this condition [3, 4]. According to the study's findings, the COVID-19 can spread between persons. As a consequence of the virus' development, there have been variations in the symptoms and transmission rates of SARS-CoV-2 throughout time. This is why it is important to identify the problem as soon as possible.

Those afflicted with COVID-19 must be separated and treated separately from the rest of the population if the illness is defeated. With the advent of diagnostic testing (current infection) and antibody tests, COVID-19 may now be diagnosed in infected individuals (past infection). Antigen tests and RT-PCR may be used to swiftly detect COVID-19 by evaluating short RNA sequences for the presence of coronaviruses, as opposed to other methods [5, 6]. False positives (FPs) are more likely to occur if you utilize antigen testing rather than RT-PCR when diagnosing a disease. It is possible that using current approaches, the detection of COVID-19 will be impeded by the following issues: RT-PCR findings that are negative for COVID-19 infection are not considered to rule out the possibility of infection. Additional inquiry is necessary to rule out the possibility of a false-negative situation [7, 8]. It is necessary to use highly specialized materials, equipment, and staff to get outcomes in a matter of hours or days.

Furthermore, early detection of the virus is critical because COVID-19-induced pneumonia is more likely to be lethal in some groups of people than in others. However, early diagnosis is not always possible or practical because of the incubation period, which may last days. These limitations need the development of novel COVID-19 detection technologies.

It is also possible to detect COVID-19 infection using a wide range of nonlaboratory methods. As part of the operation, chest X-ray data must be reviewed. Radiology journal articles have claimed that chest X-rays may be beneficial in determining whether or not a patient has pneumonia. According to the research, emphysema and chronic obstructive pulmonary disease (COPD) are life-threatening in people with COVID-19 [9]. The X-ray images of patients who have COVID-19 symptoms show some significant abnormalities such as horizontal white lines, bands, or reticular changes, as well as some visual marks like ground-glass opacities—hazy darkened spots that can distinguish those who have COVID-19 infection from those who do not, according to several additional studies [10, 11]. A chest X-ray system may be useful in identifying, measuring, and following COVID-19 patients due to this study. For example, the resolution of a CT scan has significantly enhanced. Compared to other imaging modalities, this imaging modality is less expensive and has better sensitivity than other imaging modalities, such as chest X-ray images. When testing kits and screening stations are not accessible, X-ray equipment may be used to detect COVID-19. It is also fast since it uses several cases at once.

When using the COVID-19 detection approach, which depends on X-rays, radiologists must manually inspect and delete abnormalities from each X-ray picture before using the procedure. This would need the involvement of a group of medical professionals. Patients with COVID-19 may be detected from chest X-rays in a short period and with high accuracy, making the diagnostic process easier [12]. Many healthcare institutions utilize machine learning to detect ailments and diseases [13, 14]. For example, in a patient's X-ray and computed tomography (CT) lung pictures, many supervised learning algorithms, including logistic regression, random forests, and support vector machines (SVMs), recognized it [15–18].

Deep learning systems outperform more time-consuming and labor-intensive conventional techniques for producing high-quality outcomes. This is because deep learning systems extract features on their own. Using deep learning, it is now possible to make scant contributions to interpreting medical pictures and attaining excellent classification performance with fewer time-consuming simulated workloads [19]. It is possible to train a CNN; however, the dearth of publicly accessible picture databases and the restricted quantity of patient data provide significant challenges [20].

This research used a deep convolutional neural network (CNN) model to categorize X-ray pictures into COVID-19 and healthy. There are 9803 COVID-19 photos and 8960 normal images from different public sources available as training and testing images to train and test the model. In the hopes of saving time and money, the solution created as a result of this study will enable physicians to detect disease earlier in the course of a patient's illness. If this method is perfected, it may be used in other types of medical images, including CT, MRI, and MEG, to demonstrate signature patterns in those images. In the course of this study, we may be able to detect early indicators of lung, heart, and cancer illness. This may have implications for how I treat patients who have already been diagnosed with these diseases.

The contributions of this work are summarized as follows.

1. The current work developed a more generalized model that could generate results across several COVID-19 datasets along with performance superior to the many of the previous works which was limited to one or two public datasets.
2. Proposed a cutting-edge automated COVID-19 diagnosis system based on a pretrained deep learning model to detect COVID-19 patients using chest X-ray images. The created model uses multiple residual network (ResNet) versions, spanning 34 to 101 layers.
3. Comparative analysis of the performance of the models ResNet34, ResNet50, and ResNet101 in terms of training time and accuracy. The work also investigates whether accuracy improved with complexity of the model.
4. The research also reveals a key fault in the CNN architecture: the incorrect predictions were made with 100% certainty. It is a question of the model's dependability, particularly when it comes to medical diagnosis.

The remainder of the paper is organized as follows.  Section 2 provides a review of previous literature relevant to our project. The technique is described in depth in Section 3, which contains the dataset used in this study as well as additional processes including the suggested network design and the training pipeline. A summary of the findings was presented in Section 4, followed by a discussion in Section 5. The conclusions are included in Section 6, which is followed by references.

## 2. Literature Review

A number of studies have shown that deep learning (DL) technologies are the most promising technique for dealing with disease diagnosis using medical imaging [21, 22]. The detection of the COVID-19 from X-ray, CT scan, and ultrasound images using deep learning algorithms has been the subject of much investigation [23–25]. There was no need to include previous research on other types of chest images because our investigation was solely focused on chest X-ray classification (COVID-19 or healthy).

Researchers at the University of California, Los Angeles (UCLA), have built a deep convolutional neural network model utilizing digitized chest X-rays to identify COVID-19 pneumonia patients automatically. The researchers who conducted the research published the findings in Nature Communications journalist of three publicly accessible datasets, and a GitHub repository was used in this inquiry [26]. It is possible to get chest X-ray images using one of two databases: ChestX-ray8 [27] and Chest X-Ray Images (Pneumonia). Their study discovered that the ResNet50 model outperformed the other models, with 96.1% of all models achieving the best overall performance. Train and evaluate the model throughout its development; just a small quantity of data was needed to do so (50 COVID-19 and 50 normal chest X-rays).

A new model, established by [28], was designed to predict the outcome of patients who had their X-ray pictures obtained by themselves. This study included the analysis of 100 chest X-ray pictures taken from 70 COVID-19 patients and 1431 chest X-ray images taken from pneumonia patients who did not have COVID-19. The findings were published in the journal Chest. The classification head is one of the three primary components of this paradigm. The other two components are anomaly detection and classification. Backbone networks, classification heads, and anomaly detection heads are some of the other components of the system. The core network, pretrained on ImageNet, comprises 18 layers of residual CNNs. ImageNet is the name of this massive generalized dataset for picture classification that has been created. This model has the potential to properly detect COVID-19 and non-COVID-19 patients with 96% and 70.65% accuracy, respectively.

[29] also used deep learning to identify COVID-19 patients from a limited number of chest X-ray scans, which they found effective. Their results were obtained using ResNet50, a pretrained network with an accuracy of 89.2%.

[18] have also used deep features to diagnose coronavirus illness. It was discovered in this study that utilizing ResNet50 and the support vector machine (SVM)-based model with 95.38% accuracy and 91.41% *F*1-score could be built.

The authors [30] previously used transfer learning to classify images into good and bad health using a combination of three datasets [26, 31] and from Kaggle. They found that transfer learning effectively categorized photographs into good and bad health. The researchers utilized 224 chest X-ray images of COVID-19-infected persons, 714 images of people suffering from pneumonia, and 504 images of healthy people to train their computer model. In a recent study, the researchers discovered that transfer learning might identify errors in limited medical imaging datasets. The findings demonstrate that deep learning used in X-ray imaging can consistently detect important biomarkers associated with COVID-19 disease, with a precision of 96.78%.

[32] constructed their DarkNet model in 2020 using the widely used dataset [26], which Cohen, 2020, provided. Leaky ReLU is used to activate all 17 convolution layers in their model, resulting from a bug in the algorithm. The model was trained and evaluated using 127 COVID-19 chest X-ray pictures collected from participants and 500 normal chest X-ray photos acquired from the same subjects. When it comes to binary classification, this model has an average overall accuracy of 98.08%; however, when it comes to multi-class classification, it only has an average overall accuracy of 87.02%.

According to [33], transfer learning and image augmentation were used to identify COVID-19 in chest X-ray pictures of healthy patients. COVID-19, viral pneumonia, and COVID-19 are all employed in this study to classify patients into one of two categories: I, “normal,” and II, “COVID-19,” according to the researchers. They employed 423 COVID-19 images for training and validation, 1485 images of viral pneumonia, and 1579 photos of normal chest X-rays among the images they used for training and validation. A 99.70% accuracy rate and an *F*1-score of 99.70% were achieved by this group in binary classification, indicating that they did very well.

[34] developed a deep convolutional neural network (CNN) model for classifying three forms of pneumonia: bacterial, viral, and COVID-19 pneumonia. The research is based on two datasets: [26] and “Chest X-Ray Images (Pneumonia)” [31], and achieved accuracy of 89.6% and 95%, respectively.

[35] drew on a repository hosted by Kaggle and dubbed the “COVID-19 Radiography Database,” for their research. The HSGO algorithm and the SVC classifier will construct the suggested pipeline. To establish how many COVID-19 images impacted the model's accuracy, scientists analyzed three separate sets of data to determine how many images impacted the model's accuracy. According to a further in-depth investigation, utilizing the whole dataset resulted in the maximum accuracy possible, 99.65%.

According to one research conducted by Abed and colleagues, regular machine learning models and deep learning (DL) models performed differently when automatically differentiating between healthy individuals and COVID-19-infected people in X-ray pictures. The researchers created this dataset, called COVID-19 vs. normal, which is vast in size (400 healthy cases and 400 COVID cases). Based on the results of the trials, it found the deep learning model. ResNet50 had the highest accuracy, with a score of 98.8%. However, all of the models achieved satisfactory results.

On the other hand, a CovidGAN model built by [36] used an auxiliary classier generative adversarial network (ACGAN) to generate synthetic chest X-rays (CXR) images, which was based on the ACGAN technique. They discovered that including CovidGAN's synthetic pictures into CNNs increased the performance of CNNs when it came to COVID-19 recognition. The accuracy of CNN classification was 85%, but when CovidGAN pictures were utilized to build synthetic images, the accuracy increased to 95%.

The majority of the COVID-19 research initiatives made public so far have relied on pretrained models to conduct their investigations. These models must be pretrained on larger, more broad datasets such as ImageNet before being used in the production environment. A smaller dataset was also employed to train and test the models in virtually all research, which was always beneficial.

## 3. Materials and Methods

### 3.1. Dataset

Data are at the heart of deep learning, and it is used to power these learning models. Because COVID-19 is a new disease, there is no appropriate sized dataset to use for this research. As a result, we had to acquire chest X-ray images from five distinct publicly available image databases to generate a dataset.

1. COVID-19 Radiography Database ([33, 37]).
2. GitHub repository by [26].
3. Chest X-Ray Images (Pneumonia) [31].
4. Synthetic-covid-cxr-dataset [38].
5. https://github.com/agchung/-COVID-chestxray-dataset

Some of the datasets listed above have been frequently used in past research (Table 1). All the collected images were divided into two categories: COVID and normal. We obtained 12650 images for COVID-19 class and 11787 images for normal class by integrating the images from the five publically available datasets. We transformed all of the images into a single format, .png, because they had multiple extensions like .jpg, .png, .jpeg, and so on. A total of 20% of the images obtained were used for testing. 20% of the training images were randomly allocated for validation while training the model. Because all of the photos were of different sizes, they were all reduced to 256 × 256 before being loaded into the model, significantly reducing the feature pool extracted and increasing computational speed. There was no image preprocessing to improve the quality of the images, and no augmentation to increase the quantity.

**Figure 1:**
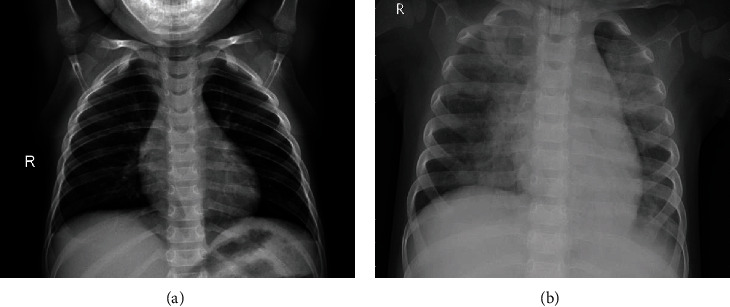
Samples of chest X-ray images from prepared dataset. (a) Normal. (b) COVID-19.

The prepared dataset for model training is summarized in Table 2 and some samples of chest X-ray images from the created dataset are shown in Figure 1. COVID-19 sufferers' lungs are spotty and cloudy, as seen in the images, when contrasted to normal, healthy lungs. Additionally, on the lower lobes and periphery of the lungs several distinctive features can also be seen [43].

### 3.2. Architecture of Deep Transfer Learning

CNNs are a type of deep neural network (DNN) used for image recognition [44]. To build a CNN model, inputs must be transformed into a CNN-friendly format. Frequently images are viewed as matrices. During training, the model tries to understand the differences between the categories, which helps it predict the labels on unknown images. CNN uses three layers to efficiently accomplish its function: convolutional, pooling, and fully connected (FC). The convolutional layer with pooling extracts the most distinct characteristics. The fully connected layers handle the classification task.

However, training the CNN from scratch takes a significant amount of time and data to achieve the requisite accuracy. Given the scarcity of available images, we chose to start with a model trained on an extensive dataset and transfer weights [45, 46]. This will eliminate the bottleneck associated with feature extraction in our CNN training. All that is required now is to adjust the final classification layer to one that is appropriate for the image classification challenge at hand. In the current work, we chose three well-established CNN architectures that have attained state-of-the-art performance and were pretrained on the ImageNet [47] dataset, which contains 1.4 million annotated images classified into 1000 classes. The chosen architectures, ResNet34, ResNet50, and ResNet101, were used for the classification of COVID-19 chest X-ray images to binary classes (COVID-19 and normal (healthy)). Additionally, we used a transfer learning technique that was implemented using the ImageNet dataset to overcome the limitations of insufficient data and training time.

**Figure 2:**
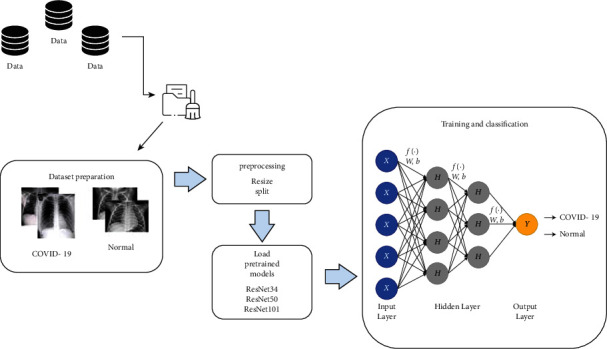
Block diagram of the proposed system.

Figure 2 shows the schematic representation of conventional CNN including pretrained ResNet34, ResNet50, and ResNet101 models for the prediction of normal (healthy) and COVID-19 classes from chest X-rays.

### 3.3. ResNet

**Figure 3:**
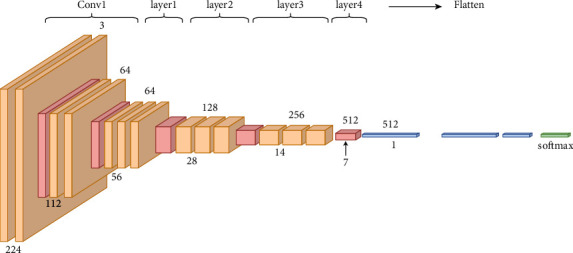
Detailed architecture of ResNet.

The residual neural network (ResNet) model [48] is an upgraded version of the convolutional neural network (CNN) (Figure 3). Unlike all other publicly available CNNs, ResNet incorporates residual (skip) connections between layers, avoiding the vanishing gradient problem that happens as the network becomes more extensive and complicated. Additionally, bottleneck blocks are employed in the ResNet model to accelerate training [49].

ResNet comes in a variety of variations that all operate on the same principle but have a variable amount of layers (Table 3). The terms ResNet34, ResNet50, and ResNet101 refer to variations that work with 34, 50, and 101 neural network layers, respectively.

### 3.4. Model Training

ResNet34, ResNet50, and ResNet101 were trained using the FAST AI package built on top of PyTorch. The training was conducted on an NVIDIA Quadro P1000 GPU with 32 GB RAM. The training begins at a learning rate of 0.001. We employed the Adam algorithm [50] as an optimizer. We originally set up the training to 100 epochs. However, if the validation loss does not constantly reduce over a lengthy period, the process will terminate prematurely. The batch size was 16 to avoid the memory exhaust. The model's performance is evaluated by applying the model to the test dataset. We used NumPy, OpenCV, scikit-learn [51], and other open-source tools for further processing and analysis.

### 3.5. Performance Metrics

The confusion matrix is a table that describes the accuracy of classification task [52]. It also shows the evaluation metrics such as true positives (TP), false positives (FP), false negatives (FN), and true negatives (TN). Each of them is defined as follows.

- True Positives (TP): These are number of images in which we predicted the class (COVID or normal), and they do belong to the predicted class.
- True Negatives (TN): We predicted that the images do not belong to a class and they actually do not belong to the class.
- False Positives (FP): We predicted a class, but the images do not actually belong to the predicted class (also known as a “type I error”).
- False Negatives (FN): We predicted that images do not belong to a class, but they actually belong (also known as a “type II error”).

The model performance metrics like accuracy (equation (1)), error rate, recall (equation (2)) precision (equation (3)), and *F*1-score (equation (4)) were derived from these data. The most commonly used fundamental measure classifiers are accuracy (ACC) and error rate (ERR) [53]

(1) $$\begin{array}{c} \text{Accuracy}=\frac{\text{TP}+\text{TN}}{\text{TP}+\text{TN}+\text{FP}+\text{FN}}, \end{array}$$

(2) $$\begin{array}{c} \text{Recall}=\frac{\text{TP}}{\text{TP}+\text{FN}}, \end{array}$$

(3) $$\begin{array}{c} \text{Precision}=\frac{\text{TP}}{\text{TP}+\text{FP}}, \end{array}$$

(4) $$\begin{array}{c} \text{F}1\text{ score}=\frac{2*\text{Precision}*\text{Recall}}{\text{Precision}+\text{Recall}}. \end{array}$$

## 4. Results

**Figure 4:**
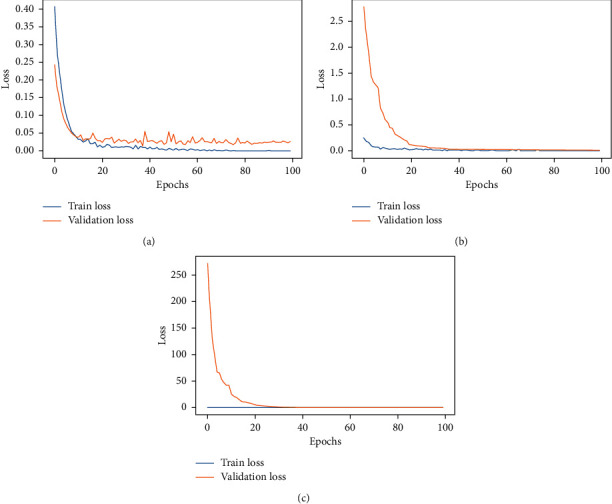
Loss for the (a) ResNet34, (b) ResNet50, and (c) ResNet101.

**Figure 5:**
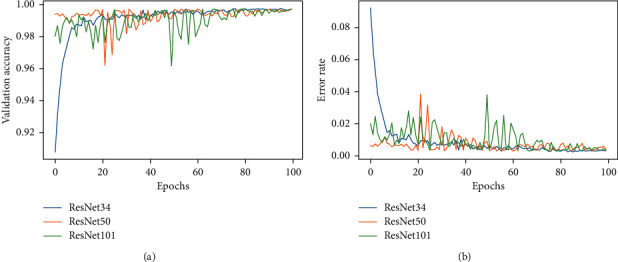
The flow graph. (a) Validation accuracy. (b) Error rate of the ResNet34, ResNet50, and ResNet101 models.

To do a COVID-19 detection using the obtained chest X-ray dataset, we developed three CNNs, ResNet32, ResNet50, and ResNet101 that were pretrained on ImageNet weights. A learning curve was used to assess the overall performance of the models. The learning curve is a mathematical depiction of the model's performance during training. The train and validation losses of the three models are presented in Figure 4. These graphs allow us to see if the models are overfit or not.  Figure 5 also depicts the validation accuracy and error rate for the three architectures that were designed. The plot of training loss decreasing to a point of stability is shown in Figure 4. Meanwhile, the validation loss plot stabilizes and has a small gap between it and the training loss. In terms of learning curves, it can be noted that the performance of ResNet34, ResNet50, and ResNet101 is astonishingly well fitted, by extracting all of the information needed for the effective classification of chest X-rays to COVID-19 or healthy (normal).

A detailed performance analysis on the validation set is used to monitor the model performance during the training process. The confusion matrix (Table 4) shows that, out of 3751 validation samples, the ResNet34 trained model predicted 12 samples to belong to a wrong class as opposed to 3739 correct predictions resulting in 99.68% validation accuracy with a 99.67% *F*1-score. It is evident that the ResNet34 outperforms the other three architectures in terms of accuracy (Table 5). The precision, recall, and *F*1-score for each class in validation samples are also shown in Table 6.

**Figure 6:**
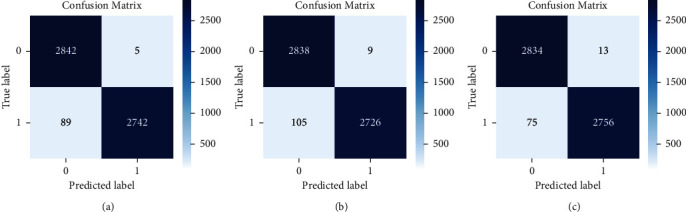
Confusion matrices: (a) ResNet34, (b) ResNet50, and (c) ResNet101 on test dataset.

**Figure 7:**
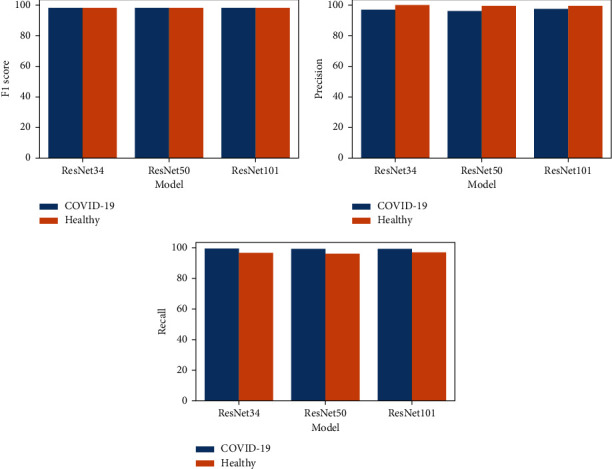
Test data performance of the models on individual classes.

Following that, we examined the performance of the pretrained model on the test data photos (Figure 6). The confusion matrices for the three ResNet model variations accessible for the experiment are shown in Figure 6, namely, ResNet34, ResNet50, and ResNet101. In all of them, the most normal (healthy) chest X-ray images were predicted as COVID-19. However, a smaller percentage of COVID-19 photos were misclassified as normal. This could be because some of the normal X-ray images possessed visual qualities that the model was unable to detect. By examining the total number of correct and incorrect predictions, and the performance of the models on individual classes (Figure 7), we may infer that ResNet101 performs the best. However, the value is not much different from the other models. Adding layers or creating a more sophisticated architecture does not always result in increased prediction accuracy [54], and here, there was only a slight performance rise with more layers. So, we can say that performance of methods is not always related to the complexity of the network. So by comparing the computational time (Table 7), which is a result of more deep layers (complexity of the network), and the model's performance, we concluded that ResNet34 would be a better choice for the chest X-ray binary classification task.

**Figure 8:**
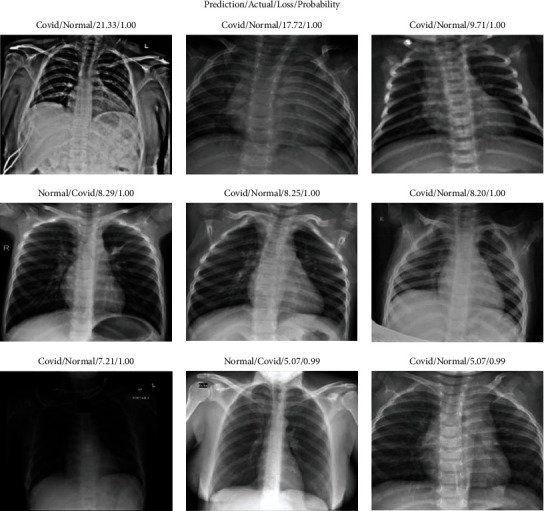
Some of the dataset's misclassified images (labeled as predicted class/actual class/loss value/probability of prediction).

When we talk about the misclassifications in Figure 7 confusion matrices, it is further evaluated in Figure 8 where most of the misclassifications occur for normal images that were wrongly predicted as COVID-19 but with high confidence.

## 5. Discussion

The effective detection of COVID-19 cases as soon as possible has emerged as a critical aspect in containing the outbreak in pandemic hot regions. Additionally, monitoring chronic coronavirus infections may aid in the prediction of new variant risks, as the virus commonly mutates under favorable conditions. It has lately been debated whether chest X-ray pictures are effective in diagnosing COVID-19, owing to the time delay and lower reliability associated with the RT-PCR or antigen testing methods [34, 55]. The current study created a fully automated diagnostic tool to classify chest X-rays into normal and COVID-19 categories based on their appearance in response to these findings. The results of this study demonstrate that an artificial intelligence system driven by a fine-tuned pretrained model, as developed in this work, can accurately anticipate the presence of COVID-19 in a chest X-ray image and distinguish it from normal conditions. Consequently, our findings improve upon prior research by emphasizing the usefulness of chest X-ray imaging in other medical diagnostic procedures.

Not only is the model's accuracy a critical research problem for us. We aimed at the factors that influence these outcomes. The current research used transfer learning to construct a COVID detection system using chest X-rays. The methodology was not unique as many previous studies had already demonstrated the utility of transfer learning or pretrained models in this classification task [18, 33]. However, the majority of them were based on a limited dataset. As a result, we cannot always state that the results are generalizable because they only reflect a small fraction of the worldwide COVID cases. We require a model that can generalize across a wide range of samples worldwide. So, we can say that the dataset created in this study is the most promising characteristic of the current research ahead of the model development.

The performance analysis of the various transfer learning methodologies suggested in this research is the next goal of the project. The ratio of correctly categorized images out of total images is the important statistic. Since we fine-tuned the pretrained model's top layers, we have already expected the best accuracy, as fine-tuning always outperforms classic feature extraction and end-to-end CNN techniques. The suggested system learnt the basic abstract features of the images from the lower layers, while the top layers learned high-level features on the target input images, resulting in high accuracy. The loss and accuracy graphs of the three versions of the residual networks are substantially identical in Figures 4 and 5. Its training impact is outstanding, and the loss has been constant. Tables 5 and 7 also reveal that the accuracy, recall, precision, and *F*1-scores of these models were comparable. This addresses two research concerns: (1) to what extent can chest X-ray binary classification (COVID vs healthy) benefit from transfer learning in deep learning with an improved dataset?, (2) how do the different transfer learning scenarios improve performance?

However, there is cause for concern. The number of parameters we need to train for each model changes depending on the number of layers. ResNet101 contains 101 layers and 44.5M parameters, as shown in Table 8, yet the detection impact is not much better than the other residual networks on the COVID-19 dataset. Table 5 illustrates the time it takes to calculate all of the models. As can be seen, increasing the number of layers and hence the parameters elongates the training time. The longer it takes to extract features, the more complicated the network structure is, but this does not affect accuracy. Compared to more complex versions of ResNet, ResNet34 requires the least amount of time to train and obtain a comparable performance.

### 5.1. Performance Comparison

The table gives a performance comparison of the previous works with the same problem with the proposed models. We compare our research with recent work, as shown in Table 9. [57] used seven customized deep CNN models to classify COVID-19 and healthy people. The most performance occurred for VGG19 and DenseNet201 with accuracy, F1-score, precision, and recall of 90%, 91.5%, and 90%, respectively. [58, 59] used the transfer learning methods to achieve accuracies up to 96% and 91.62%, respectively.

[32] with their customized DarkNet model achieved high performance of 98.08% accuracy, which is closer to our model. Even though majority of models achieved good performance, the only issue was that many of them used one or two datasets for their works which kept the model generalization capability in question. But our model outperforms these works in terms of accuracy and the quantity of the data it handles.

The organization of the previous work [34] is similar to ours; they used the Xception model, which, like ours, makes use of residual connections to address the issue of disappearing gradients. [59] also employed ResNet50v2 in their work. However, our objective here was to construct a more accurate model and develop a model that was much more generalizable to a much larger dataset than in these previous publications; then only, it can be suitable for real-life scenarios. Additionally, [34] compared the number of parameters in their model to those in prior publications. However, we analyzed the number of parameters in various ResNet versions generated for the COVID-19 identification task in our work. We attempted to develop a trade-off between the number of parameters in the model (training time) and its accuracy, which was essentially a comparison of training time and accuracy. We also included the prediction visuals in the discussion section as in [34]. However, they were utilized not only to illustrate the predictions of the model but also to determine whether the predictions were correct or incorrect and the level of confidence in those predictions. Our work attempted to deduce a significant inaccuracy in the CNN and will have a detrimental effect on the medical diagnosis process.

The misclassifications that occurred in the test dataset are depicted in Figure 6. It is clear that the healthy images had more mistakes because they were anticipated as COVID-19. Figure 8 depicts some of the dataset's misclassified images. However, it is worth noting that these incorrect forecasts were forecasted with a probability of 99% or 100% (confidence). This highlights a significant CNN flaw. CNNs are not without faults, even when they have great accuracy. The error bars associated with a CNN's forecast are unavailable. [61] already discussed how deep neural networks can be easily fooled and produce high confidence predictions based on rubbish images. So, in a domain, particularly in the medical domain, understanding the mistake associated with the prediction is critical since the judgments affect people's lives. So as part of our future study, we plan to look into ways to provide error bars for CNN predictions.

## 6. Conclusions

The paper presented a deep learning model for detecting COVID-19 incidence from chest X-rays. The model is trained with a prepared dataset containing images from varied public sources containing chest X-rays of healthy and COVID categories. The model's performance evaluation for image classification using transfer learning yields a classification accuracy of above 98%. The current research also presented a detailed study of the performance of the three distinct residual network models with numbers of layers ranging from 34 to 101. It was evident that the training and validation loss plots for all specified architectures showed the least gap regardless of the number of layers. This means that all models could achieve an optimum value of weights with a decent degree of accuracy. However, the more complex model required more training time to attain the best results. ResNet34 was the best model since it could reach a 98.34% accuracy with a small amount of training time. The proposed model generalized well to the dataset, making it a better candidate for real-time applications than state-of-the-art models developed with a smaller dataset. Furthermore, we discovered that more healthy images were predicted to be of the COVID class with high confidence. This unravels a significant flaw in CNN: CNN has a reliability issue that hugely impacts medical decision-making. As a result, we planned to address this issue in future work, including COVID prediction from chest X-rays.

## Figures and Tables

**Table 1:** List of some of the benchmark datasets used in previous works.

Database	Previous works
COVID-19 Radiography Database https://www.kaggle.com/tawsifurrahman/covid19-radiography-database [33, 37]	[34, 39, 40]
GitHub repository by [26] https://github.com/ieee8023/covid-chestxray-dataset/tree/master/images	[41]
Chest X-Ray Images (Pneumonia) https://www.kaggle.com/paultimothymooney/chest-xray-pneumonia [36]	[34, 42]

**Table 2:** Distribution of the collected images.

	Category	No. of images
Train	COVID-19	9803
	Normal	8956

Test	COVID-19	2847
	Normal	2831

**Table 3:** Sizes of outputs and convolutional kernels for ResNet versions.

Layer name	Output size	34 layers	50 layers	101 layers
conv 1	112 × 112	7 × 7, 64, stride 2		
conv 2.x	56 × 56	3 × 3 max pool, stride 2		
		$\left[\begin{array}{c} 3x3,64\\ 3x3,64 \end{array}\right]x3$	$\left[\begin{array}{c} 1x1,64\\ 3x3,64\\ 1x1,256 \end{array}\right]x3$	$\left[\begin{array}{c} 1x1,64\\ 3x3,64\\ 1x1,256 \end{array}\right]x3$
conv 3.x	28 × 28	$\left[\begin{array}{c} 3x3,128\\ 3x3,128 \end{array}\right]x4$	$\left[\begin{array}{c} 1x1,128\\ 3x3,128\\ 1x1,512 \end{array}\right]x4$	$\left[\begin{array}{c} 1x1,128\\ 3x3,128\\ 1x1,512 \end{array}\right]x4$
conv 4.x	14 × 14	$\left[\begin{array}{c} 3x3,256\\ 3x3,256 \end{array}\right]x6$	$\left[\begin{array}{c} 1x1,256\\ 3x3,256\\ 1x1,1024 \end{array}\right]x6$	$\left[\begin{array}{c} 1x1,256\\ 3x3,256\\ 1x1,1024 \end{array}\right]x23$
conv 4.x	7 × 7	$\left[\begin{array}{c} 3x3,512\\ 3x3,512 \end{array}\right]x3$	$\left[\begin{array}{c} 1x1,512\\ 3x3,512\\ 1x1,2048 \end{array}\right]x3$	$\left[\begin{array}{c} 1x1,512\\ 3x3,512\\ 1x1,2048 \end{array}\right]x3$
	1 × 1	Average pool, 1000-d fc, softmax		
FLOPs		3.6 × 10^9^	3.8 × 10^9^	7.6 × 10^9^

**Table 4:** Validation confusion matrix.

Models		True:COVID-19	True: healthy
ResNet34	Predicted: COVID-19	1924	8
	Predicted: healthy	4	1815

ResNet50	Predicted: COVID-19	1923	11
	Predicted: healthy	5	1812

ResNet101	Predicted: COVID-19	1925	10
	Predicted: healthy	3	1813

**Table 5:** Validation performance metric.

Models	Accuracy (%)	Recall (%)	Precision (%)	*F*1-score (%)
ResNet34	**99.68**	**99.56**	99.78	**99.67**
ResNet50	99.57	99.40	99.72	99.56
ResNet101	99.65	99.45	**99.83**	99.64

**Table 6:** Validation classification report.

Models		Recall (%)	Precision (%)	*F*1-score (%)
ResNet34	COVID-19	99.79	99.59	99.69
	Healthy	99.56	99.78	99.67

ResNet50	COVID-19	99.74	99.43	99.59
	Healthy	99.40	99.72	99.56

ResNet101	COVID-19	99.84	99.48	99.66
	Healthy	99.45	99.83	99.64

**Table 7:** Test data performance metric.

Models	Accuracy (%)	Recall (%)	Precision (%)	*F*1-score (%)	Training time
ResNet34	**98.34**	**96.86**	99.81	**98.31**	**609 minutes**
ResNet50	97.99	96.29	99.67	97.95	1200 minutes
ResNet101	98.45	97.35	**99.53**	98.42	1700 minutes

**Table 8:** Number of parameters in ResNet versions [56].

ResNet version	Number of parameters (in millions)
ResNet34	21.8
ResNet50	25.6
ResNet101	44.5

**Table 9:** Performance comparison with previous works.

Works	Architecture	Accuracy (%)
[57]	VGG19 DenseNet201	90
[58]	COVIDPEN	96
[59]	DenseNet201 + ResNet50V2 + Inceptionv3	91.62
[60]	VGG19, ResNet152 Xception, DenseNet201 InceptionResNetV2	96
[32]	Customized DarkNet	98.08
Our model	ResNet34	98.34

## Data Availability

The dataset used in this study is available publicly at https://www.kaggle.com/tawsifurrahman/covid19-radiography-database, https://github.com/ieee8023/covid-chestxray-dataset/tree/master/images, and https://www.kaggle.com/paultimothymooney/chest-xray-pneumonia.
